# Longitudinal study on the association between three dietary indices, anthropometric parameters and blood lipids

**DOI:** 10.1186/s12986-015-0042-1

**Published:** 2015-11-19

**Authors:** Evelien Mertens, Benedicte Deforche, Patrick Mullie, Johan Lefevre, Ruben Charlier, Sara Knaeps, Inge Huybrechts, Peter Clarys

**Affiliations:** Faculty of Physical Education and Physiotherapy, Department of Human Biometrics and Biomechanics, Vrije Universiteit Brussel, Pleinlaan 2, 1050 Brussels, Belgium; Erasmus University College, Laerbeeklaan 121, 1090 Brussels, Belgium; International Prevention Research Institute (iPRI), 15 chemin du Saquin, Ecully, Lyon, France; Faculty of Kinesiology and Rehabilitation Sciences, Department of Kinesiology, KU Leuven, Tervuursevest 101, 3001 Leuven, Belgium; International Agency for Research on Cancer (IARC), Dietary Exposure Assessment Group (DEX), 150 Cours Albert Thomas, 69372 Lyon, France; Faculty of Medicine and Health Sciences, Department of Public Health, Ghent University, De Pintelaan 185, 9000 Ghent, Belgium; Department of Movement and Sports Sciences, Ghent University, Watersportlaan 2, 9000 Ghent, Belgium

**Keywords:** Waist circumference, Body mass index, Blood cholesterol, Diet quality index, Mediterranean diet score, Healthy eating index-2010

## Abstract

**Background:**

From a health promotion perspective, the use of dietary indices is preferred above single nutrients and foods to evaluate diet quality. Longitudinal research about the association between dietary indices and respectively anthropometric parameters and blood lipids is lacking. The aim of this study was to investigate the longitudinal association between three dietary indices (Healthy Eating Index-2010 (HEI), Mediterranean Diet Score (MDS) and Diet Quality Index (DQI)) and respectively anthropometric parameters and blood lipids.

**Methods:**

A three day diet record was completed by 373 men and 197 women in 2002–2004 and 2012–2014. HEI, MDS and DQI were calculated. Waist circumference (WC) and Body Mass Index (BMI) were used as anthropometric parameters. A linear regression analysis was performed to investigate associations between changes in dietary indices and changes in respectively anthropometric parameters and blood lipids, adjusted for potential confounders.

**Results:**

Only in men an increase in all three dietary indices was associated with a decrease in WC and BMI in the non-adjusted analysis and for HEI and DQI also in the adjusted analysis. No longitudinal associations were found between dietary indices and blood lipids both in men and women.

**Conclusions:**

Only few associations were found between dietary indices and anthropometric parameters, whilst no associations were found with blood lipids. An increase in dietary indices was associated with an improvement in anthropometric parameters only in men. As this is the first study investigating associations between changes in dietary indices and changes in respectively anthropometric parameters and blood lipids, further research is needed to evaluate these possible associations.

## Background

Cardiovascular disease (CD) is the main cause of death in women in all European countries and in men in all but six countries [1]. Anthropometric parameters and blood lipids are critical cardiovascular health indicators: a high waist circumference (WC) and Body Mass Index (BMI) and a deteriorated blood lipid profile are associated with high incidence rates of CD [2]. Although there is evidence supporting a causal link between dietary factors and CD [3], the contribution of overall diet quality is insufficiently investigated so far. The use of dietary indices as a measure of diet quality has emerged to be a preferred approach to study the relation between nutrition and chronic diseases, since food is mostly consumed in combination rather than separately and interactions between nutrients are possible [4, 5]. As a consequence nationally and internationally accepted dietary indices have been developed to measure diet quality, each having specific principles and approaches in its concept and calculation. The Healthy Eating Index-2010 (HEI) is based on dietary guidelines summarized in the United States Department of Agriculture Food Guide Pyramid [6]. The Mediterranean Diet Score (MDS) is based on the adherence to the Mediterranean diet [7]. The Diet Quality Index (DQI) evaluates the adherence to Flemish food-based dietary guidelines [8]. The HEI and the DQI are calculated by comparing the participants’ dietary intake with prior defined guidelines, while the MDS uses collected data to obtain gender-specific median component intakes.

In cross-sectional research, the HEI was negatively associated with overweight and obesity [9, 10], but weakly with blood lipids [11–13]. The cross-sectional association between the MDS and blood lipids remains equivocal [14–18]. The DQI showed a positive cross-sectional association with triglycerides in the unadjusted model [19]. To disentangle the complex relationship between diet quality and health-related parameters, it is critical to study the longitudinal associations between diet quality as measured by dietary indices and respectively anthropometric parameters and blood lipids. A longitudinal study showed that baseline diet quality as measured by the MDS, the DQI and the Higher Ideal Diet Index did not predict mortality risk over a follow-up period of 12 years [20]. Unfortunately little is known about stability of dietary indices. This knowledge may provide valuable information for longitudinal research which associated a measure of diet quality at baseline with health parameters at follow-up [20].

Since it has been demonstrated that age [21] and smoking behavior [22] may have an influence, these potential confounders should be taken into account when studying the association between diet quality and respectively anthropometric parameters and blood lipids. Equally, cardiorespiratory fitness is considered a potential confounder in the association between diet and cardiovascular health [23]. Because of the gender-specific differences in dietary indices [24], anthropometric parameters [25] and blood lipids [26] and the complex interrelationships between those parameters, analyses should be stratified by gender.

The present study focused on three dietary indices, namely the HEI [6], the MDS [7] and the DQI [8]. The aim of this study was to investigate the longitudinal association between these three dietary indices and respectively anthropometric parameters (WC and BMI) and blood lipids (total cholesterol (TC), high-density lipoprotein (HDL) cholesterol, low-density lipoprotein (LDL) cholesterol, ratio total/HDL cholesterol and triglycerides). To take into account the influence of the above mentioned confounders, an unadjusted model and an adjusted model was developed.

## Methods

### Subjects

This study is based on data collected by the Flemish Policy Research Centre Sport, Physical Activity and Health [27]. One of the aims of this Research Centre was to examine the relationship between health behavior, physical health, mental health and physical fitness among an adult population. Therefore 46 Flemish municipalities were randomly selected by the National Institute of Statistics. Within those municipalities, a random sample of men and women between 18 and 75 years old was selected in 2002. The sample can be considered as sufficiently representative for geographic distribution, age, gender and educational level. The first test moment took place during 2002–2004, the second during 2012–2014. Of the original 1569 volunteers who participated in 2002–2004, 652 returned for retesting in 2012–2014. Of the retested sample 570 participants (men = 373, women = 197) completed the three day diet record at both measure points. All participants received information about the tests and measures before participation and signed an informed consent. The study was approved by the ethical and medical committee of the KU Leuven.

### Tests and measures

#### Dietary assessment

Participants completed a three-day diet record, in which they recorded all foods and drinks during two weekdays and one weekend day [28]. Participants were asked to weigh the amount of foods and drinks consumed if possible. Otherwise they were inquired to estimate the amount of foods and drinks consumed by using standard household measures. Information about the diet record was included in the three-day record booklet. The diet records were analyzed using Becel Nutrition software (Unilever Co.; Rotterdam, The Netherlands). Total energy intake (in kcal/day), consumption of food groups (in g/day), macronutrients (in g/day) and micronutrients (in mg/day or μg/day) were calculated.

### Healthy eating index-2010

The HEI is a measure of diet quality which is based on 12 components, including nine adequacy and three moderation components [6]. The adequacy components are total fruit, whole fruit, total vegetables, greens and beans, whole grains, dairy, total protein foods, seafood and plant proteins and fatty acids. The moderation components are refined grains, sodium and empty calories. This diet index applies a density approach to set standards, e.g. as a percentage of calories or per 100 calories, and uses least-restrictive standards. The 12 components were computed to a score of 100. A higher score indicates higher adherence. The HEI is a reliable and valid measure of diet quality [29].

### Mediterranean diet score

The MDS was computed using gender-specific median of intakes [7]. For beneficial components according to the MDS such as vegetables, legumes, fruits and nuts, cereal and fish, consumption below the median was assigned a value of 0, while consumption above the median was assigned a value of 1. For detrimental components according to the MDS such as meat, poultry and dairy products, consumption below the median was assigned a value of 1, while consumption above the median received a value of 0. Men with a consumption of ethanol between 10 and 50 g per day and women with a consumption of ethanol between 5 and 25 g per day received a value of 1; other intakes for ethanol received a value of 0. For fat intake the ratio mono-unsaturated/saturated fatty acids was determined. A value above the median was rated 1, below the median 0. Total MDS was calculated as the sum of the nine above mentioned component scores. A higher score indicates higher adherence. The MDS is a reliable and valid measure of diet quality [7].

### Diet quality index

The DQI is based on Flemish food-based dietary guidelines, which are presented in a food triangle. The food triangle consists of food groups that may be consumed in a certain amount according to their place in the pyramid. Foods in the bottom of the pyramid are healthy foods and should be consumed in higher amounts compared to products higher in the pyramid [30]. The calculation of the DQI is presented in Table 1 [8]. The Flemish food-based dietary guidelines are mostly in line with dietary guidelines of other countries [30]. This index showed good reproducibility and validity [31].

**Table 1:** Description of the Diet Quality Index (DQI) and its components

Dietary diversity
Expresses the degree of variaton in the diet (whether the subject used foods from the different food groups (FG) recommended in the Flemish food-based dietary guidelines) = (# different FG from main FG from which at least one serving was consumed)/total # main FG x 100 %
Dietary quality
Expresses whether the subject made the optimal quality choices
All food amounts were multiplied with a factor:
1 for items of the ‘preference’ food category
0 for items to be consumed with moderation from the ‘intermediate’ food category
−1 for items from ‘low-nutritious, energy-dense group’ (high caloric, but low nutrient density)
This was summed and divided by the total food amount consumed:
∑ (factor food item x food quantity food item)/∑ food quantity food item
Dietary equilibrium
Expresses the equilibrium/balance of food intakes = ∑_1_^#FG^(dietary adequacy FG—dietary excess FG)/# different FG x 100 % (see rules in adequacy and excess score below)
Dietary adequacy
Expresses the percentage of minimum recommended food intake actually consumed for all main FG = ∑_1_^#FG^(actual intake FG/min recommendation FG)/# different FG x 100 %
With the actual intake being truncated to the minimum recommended intake if exceeding the minimum recommended intake
Dietary excess
Expresses the percentage of intake exceeding the upperlevel of the recommendation = ∑_1_^#FG^((actual intake FG—upper level FG/upper level FG)/# different FG x 100 %
With the excess of a FG being truncated at 100 % when exceeding 100 % and at 0 when below 0 %
Meal index
Expresses the frequency of consumption of a breakfast, lunch and dinner per day (frequency breakfast/day + frequency lunch/day + frequency dinner/day)/3 x 100 %
Physical activity index (PA index)
Expresses the compliance with the physical activity recommendation (e,g, 30 min in moderate to vigorous phyical activity per day)
(total time spent in moderate to vigorous physical activity per day)/30 x 100 %
With the PA index being truncated at 100 % when exceeding 100 %
Total DQI = (dietary diversity score + dietary quality score + dietary equilibrium score + meal index + PA index)/5
Expresses the compliance of the subject with the Flemish food-based dietary guidelines (higher compliance gives higher DQI score)

Main/essential food groups included in the Flemish food-based dietary guidelines are beverages milk, milk products and cheese; meat, poultry and fish; and fats and oils

#### Smoking behavior

Smoking behavior was assessed using the WHO Monica Smoking Questionnaire [32]. The participants were classified into actual smokers or non-smokers.

#### Anthropometric parameters

Anthropometry was accomplished by trained people using standardized techniques and equipment as proposed by the International Society for the Advancement of Kinanthropometry [33]. Participants were measured barefoot and in minimal clothing. WC was measured using a metal tape (Rosscraft, Surrey, BC, Canada) at the narrowest level between lowest ribs and iliac crests to the nearest 0.1 cm. Body weight was measured to the nearest 0.1 kg with a digital weight scale (Seca 841, Seca GmbH, Hamburg, Germany) and body height with a stadiometer (Holtain, Crymych, UK) to the nearest 0.1 cm. BMI was calculated using the following formula: BMI = body weight (kg)/(height (m))^2^.

#### Blood samples

Participants were inquired to fast from 11:00 p.m. the evening before they were visiting the laboratory. A fasting blood sample from an antecubital vein in supine position was taken. A tube of 10 ml Venoject was taken to determine TC, HDL cholesterol, LDL cholesterol and triglycerides. Triglycerides were analyzed using the lipase/glycerol kinase/glycerol phosphate oxidase enzymatic method. HDL cholesterol was analyzed using the homogeneous polyanion/cholesterol esterase/oxidase enzymatic method. Triglycerides and HDL cholesterol were measured on an Olympus AU5400 analyzer (Olympus Diagnostica, Hamburg, Germany). LDL cholesterol was calculated using the following formula: LDL cholesterol = TC – HDL cholesterol – triglycerides/5 [34].

#### Cardiorespiratory fitness

Cardiorespiratory fitness was measured using a maximal exercise test on an electrically braked Lode Excalibur cycle ergometer (Lode, Groningen, The Netherlands). A standardized exercise protocol was used. The test started with a workload of 20 Watt, which increased with 20 Watt per minute. Participants were asked to cycle at about 70 rpm. The test leader encouraged them to reach their level of exhaustion. A Cortex Metalyser 3B Analyzer (Cortex Biophysic GmbH, Leipzig, Germany) was used to measure directly oxygen consumption with breath-by-breath respiratory gas exchange analysis. This method has proven to generate highly reliable results [35]. Cardiorespiratory fitness was assessed as peak oxygen uptake normalized for body weight (VO_2peak_ in ml/kg/min).

#### Statistical analysis

SPSS 21.0 (SPSS Inc. Chicago, IL) statistics software was used for data analysis. A drop-out analysis was performed by comparing baseline results between the follow-up group and the group that only participated in 2002–2004 using an independent samples t-test. A paired samples t-test was used to examine if there are any differences in parameters between the two test moments. Differences in actual smokers between the two test moments were investigated using the chi-square test.

Residual change scores of the dietary indices (HEI, MDS and DQI), anthropometric parameters (WC and BMI), blood lipids (TC, HDL cholesterol, LDL cholesterol, ratio total/HDL cholesterol, triglycerides), smoking and VO_2peak_ between the two test periods were calculated. Residual change scores were created by regressing the follow-up measures onto their respective baseline measures. The residualized change scores can be interpreted as the amount of change between the first and second test moment, independent of baseline levels and are preferred above simple change scores because they eliminate auto-correlated error and regression to the mean effects [36].

Associations between changes in the dietary indices and changes in respectively anthropometric parameters and blood lipids were tested in an unadjusted model and an adjusted model using multivariate linear regression with anthropometric parameters and blood lipids as continuous dependent variable. In the adjusted model analyses were corrected for the potential confounding factors age and changes in smoking and VO_2peak_ and also WC for the association with blood lipids. The analyses were stratified by gender. A two-sided 0.05 level of significance was defined.

## Results

Differences in baseline fitness components between the present sample and those who dropped-out are presented in Table 2. In women, the follow-up sample scored significantly better on BMI, WC, VO_2peak_ and HDL cholesterol compared to women who dropped-out. In men, the follow-up sample scored significantly better on ratio total/HDL cholesterol and triglycerides compared to men who dropped-out.

**Table 2:** Drop-out analysis

	Men			Women		
	Drop-out (*N* = 503)	Follow-up (*N* = 420)		Drop-out (*N* = 414)	Follow-up (*N* = 232)	
	Mean (SD)	Mean (SD)	*p*	Mean (SD)	Mean (SD)	*p*
Body Mass Index (kg/m^2^)	25.67 (3.43)	25.36 (2.72)	0.136	24.57 (4.18)	23.43 (3.03)	<0.001
Waist circumference (cm)	89.50 (10.37)	89.10 (8.50)	0.526	77.94 (9.95)	75.75 (7.49)	0.003
VO_2peak_ relative (ml/kg/min)	36.88 (9.03)	37.58 (8.18)	0.262	27.20 (6.34)	29.00 (5.80)	0.001
Total cholesterol (mg/dl)	209.07 (41.37)	205.86 (37.42)	0.240	203.31 (36.42)	208.25 (38.70)	0.126
HDL cholesterol (mg/dl)	54.35 (12.48)	55.68 (11.86)	0.114	65.71 (14.82)	70.63 (15.59)	<0.001
LDL cholesterol (mg/dl)	130.28 (36.52)	127.72 (33.86)	0.295	118.50 (31.95)	118.53 (35.18)	0.992
Ratio Total/HDL cholesterol	4.01 (1.10)	3.84 (1.02)	0.024	3.21 (0.81)	3.07 (0.90)	0.057
Triglycerides (mg/dl)	122.40 (84.06)	111.39 (63.32)	0.028	93.88 (43.09)	93.55 (40.29)	0.921

In Table 3 characteristics of participants are presented, as well as test results of the first (2002–2004) and second (2012–2014) assessment. The mean (SD) age at the first assessment was 47.07 (10.29) years in men and 45.18 (8.27) years in women. At the second assessment the mean (SD) age was 57.62 (10.30) years in men and 55.67 (8.23) years in women. In men, WC, BMI, ratio total/HDL cholesterol, DQI and HEI significantly increased with increasing age, while VO_2peak_, HDL cholesterol and actual smokers significantly decreased. There were no statistically significant differences in TC, LDL cholesterol, triglycerides and MDS between the two assessments in men. In women, WC, BMI, TC, LDL cholesterol, ratio total/HDL cholesterol, DQI and HEI significantly increased with increasing age, while VO_2peak_ and number of actual smokers significantly decreased. There were no statistically significant differences in HDL cholesterol, triglycerides, and MDS between the two assessments in women.

**Table 3:** Characteristics of the participants

	Men (*N* = 373)			Women (*N* = 197)		
	2002–2004	2012–2014		2002–2004	2012–2014	
	Mean (SD)	Mean (SD)	*p*	Mean (SD)	Mean (SD)	*p*
Age (years)	47.07 (10.29)	57.62 (10.30)	<0.001	45.18 (8.27)	55.67 (8.23)	<0.001
Waist circumference (cm)	89.04 (8.54)	90.01 (8.81)	<0.001	75.65 (7.32)	77.81 (8.39)	<0.001
Body Mass Index (kg/m^2^)	25.36 (2.72)	25.71 (3.00)	<0.001	23.43 (3.03)	24.07 (3.38)	<0.001
VO_2peak_ relative (ml/kg/min)	38.37 (7.99)	36.84 (8.63)	<0.002	29.42 (5.92)	27.93 (5.59)	<0.001
Total cholesterol (mg/dl)	206.39 (37.46)	205.74 (38.67)	0.732	207.72 (38.47)	224.55 (36.81)	<0.001
HDL cholesterol (mg/dl)	55.68 (11.87)	53.97 (11.64)	<0.001	70.80 (15.49)	69.19 (15.15)	0.100
LDL cholesterol (mg/dl)	127.95 (33.87)	130.29 (34.33)	0.166	118.13 (35.23)	136.17 (32.15)	<0.001
Ratio Total/HDL cholesterol	3.86 (1.03)	3.98 (1.18)	0.011	3.05 (0.88)	3.37 (0.85)	<0.001
Triglycerides (mg/dl)	111.73 (63.80)	108.13 (74.42)	0.347	92.02 (36.90)	95.89 (53.61)	0.285
Diet Quality Index (%)	61.98 (8.78)	63.82 (8.32)	<0.001	67.24 (8.11)	69.05 (7.07)	0.001
Mediterranean Diet Score (score on 9 points)	3.86 (1.53)	3.93 (1.54)	0.550	3.80 (1.51)	4.01 (1.47)	0.116
Healthy Eating Index-2010 (%)	44.85 (10.87)	46.68 (9.79)	0.002	49.22 (10.27)	52.03 (10.81)	0.001
	%	%	*p* chi^2^	%	%	*p* chi^2^
Actual smokers (%)	13.7	8.8	<0.001	13.1	8.5	<0.001

Table 4 represents the multivariate linear regression analysis with changes in respectively anthropometric parameters and blood lipids as dependent variable and changes in HEI as independent variable. Both in the unadjusted model (model 1) and in the adjusted model (model 2) a mean increase in HEI was associated with a decrease in WC and BMI in men. In women, no longitudinal associations were found between the HEI and anthropometric parameters. In the unadjusted model (model 1) a mean increase in HEI was associated with a decrease in LDL cholesterol in men. No longitudinal associations were found between the HEI and TC, HDL cholesterol, ratio total/HDL cholesterol and triglycerides in the unadjusted model (model 1) and with LDL cholesterol in the adjusted model (model 2) both in men and women and with LDL cholesterol in the unadjusted model (model 1) in women.

**Table 4:** Associations between changes in Healthy Eating Index-2010 and changes in respectively anthropometric parameters (waist circumference, Body Mass Index) and blood lipids (total cholesterol, HDL cholesterol, LDL cholesterol, ratio total/HDL cholesterol, triglycerides)

	Men (*N* = 373)				Women (*N* = 197)			
	Model 1		Model 2		Model 1		Model 2	
	β	R^2^	β	R^2^	β	R^2^	β	R^2^
Healthy Eating Index-2010-Waist circumference	−0.160**	0.026	−0.167**	0.225	−0.039	0.002	0.072	0.129
Healthy Eating Index-2010-Body Mass Index	−0.172**	0.030	−0.187***	0.290	−0.064	0.004	0.027	0.153
Healthy Eating Index-2010-Total cholesterol	−0.095	0.009	−0.079	0.067	−0.011	0.000	−0.081	0.078
Healthy Eating Index-2010-HDL cholesterol	0.007	0.000	−0.067	0.191	−0.010	0.000	−0.042	0.082
Healthy Eating Index-2010-LDL cholesterol	−0.107*	0.011	−0.082	0.071	0.035	0.001	−0.037	0.119
Healthy Eating Index-2010-Ratio Total/HDL cholesterol	−0.073	0.005	−0.012	0.155	−0.008	0.000	−0.059	0.132
Healthy Eating Index-2010-Triglycerides	−0.015	0.000	0.044	0.071	−0.124	0.015	−0.124	0.064

*p < 0.05, **p < 0.01, ***p < 0.001

Model 1: unadjusted

Model 2: adjusted for age, residual change score smoking, residual change score VO_2peak_relative and residual change waist circumference for the association with blood lipids

The multivariate linear regression analysis with changes in respectively anthropometric parameters and blood lipids as dependent variable and changes in MDS as independent variable is shown in Table 5. In the unadjusted model (model 1) a mean increase in MDS was associated with a decrease in WC and BMI in men. No longitudinal associations were found between the MDS and anthropometric parameters in women. No longitudinal associations were found between the MDS and blood lipids both in men and women.

**Table 5:** Associations between changes in Mediterranean Diet Score and changes in respectively anthropometric parameters (waist circumference, Body Mass Index) and blood lipids (total cholesterol, HDL cholesterol, LDL cholesterol, ratio total/HDL cholesterol, triglycerides)

	Men (*N* = 373)				Women (*N* = 197)			
	Model 1		Model 2		Model 1		Model 2	
	β	R^2^	β	R^2^	β	R^2^	β	R^2^
Mediterranean Diet Score-Waist circumference	−0.157*	0.025	−0.025	0.139	0.005	0.000	−0.132	0.198
Mediterranean Diet Score-Body Mass Index	−0.174*	0.030	−0.078	0.171	−0.033	0.001	−0.143	0.261
Mediterranean Diet Score-Total cholesterol	−0.031	0.001	−0.024	0.062	0.036	0.001	0.042	0.073
Mediterranean Diet Score-HDL cholesterol	−0.014	0.000	0.023	0.187	0.049	0.002	0.024	0.081
Mediterranean Diet Score-LDL cholesterol	−0.023	0.001	−0.051	0.067	−0.008	0.000	0.022	0.118
Mediterranean Diet Score-Ratio Total/HDL cholesterol	−0.041	0.002	−0.051	0.157	−0.041	0.002	−0.010	0.129
Mediterranean Diet Score-Triglycerides	0.021	0.000	0.063	0.073	0.026	0.001	0.003	0.049

*p < 0.05, **p < 0.01, ***p < 0.001

Model 1: unadjusted

Model 2: adjusted for age, residual change score smoking, residual change score VO_2peak_ relative and residual change waist circumference for the association with blood lipids

The multivariate linear regression analysis with changes in respectively anthropometric parameters and blood lipids as dependent variable and changes in DQI as independent variable is presented in Table 6. Both in the unadjusted model (model 1) and in the adjusted model (model 2) a mean increase in DQI was associated with a decrease in WC and BMI in men. No longitudinal associations were found between the DQI and anthropometric parameters in women. No longitudinal associations were found between the DQI and blood lipids both in men and women.

**Table 6:** Associations between changes in Diet Quality Index and changes in respectively anthropometric parameters (waist circumference, Body Mass Index) and blood lipids (total cholesterol, HDL cholesterol, LDL cholesterol, ratio total/HDL cholesterol, triglycerides)

	Men (*N* = 373)				Women (*N* = 197)			
	Model 1		Model 2		Model 1		Model 2	
	β	R^2^	β	R^2^	β	R^2^	β	R^2^
Diet Quality Index-Waist circumference	−0.138*	0.019	−0.140*	0.217	−0.018	0.000	0.076	0.129
Diet Quality Index-Body Mass Index	−0.135**	0.018	−0.129*	0.272	−0.016	0.000	0.049	0.154
Diet Quality Index-Total cholesterol	−0.086	0.007	−0.024	0.062	−0.045	0.002	−0.057	0.075
Diet Quality Index-HDL cholesterol	0.057	0.003	0.018	0.187	−0.004	0.000	0.016	0.080
Diet Quality Index-LDL cholesterol	−0.096	0.009	−0.062	0.069	−0.026	0.001	−0.049	0.120
Diet Quality Index-Ratio Total/HDL cholesterol	−0.097	0.009	−0.027	0.155	−0.016	0.000	−0.063	0.132
Diet Quality Index-Triglycerides	−0.024	0.001	0.068	0.074	−0.056	0.003	−0.039	0.051

*p < 0.05, **p < 0.01, ***p < 0.001

Model 1: unadjusted

Model 2: adjusted for age, residual change score smoking, residual change score VO_2peak_relative and residual change waist circumference for the association with blood lipids

## Discussion

This study investigated associations between changes in three dietary indices and changes in respectively anthropometric parameters and blood lipids over a 10-year follow-up period in adults. In general only few associations were found between dietary indices and anthropometric parameters in men, whilst no associations were found with blood lipids both in men and women.

The results can be summarized in three main findings. First, there were gender differences in the longitudinal associations between dietary indices and anthropometric parameters, with associations found only in men. The lack of longitudinal associations between dietary indices and anthropometric parameters in women can possibly be attributed to the health status of these participants. In women, volunteers who participated again scored significantly better on anthropometric parameters compared to those who dropped-out. Furthermore the change scores may have been too small to detect longitudinal associations. The influence of other factors such as hormone use and menopausal status may also explain the lack of associations between changes in dietary indices and changes in anthropometric parameters in women [37], however this information was not available.

The second main finding was that there were no longitudinal associations between dietary indices and blood lipids in both genders. Research concerning the response of blood lipids to changes in diet is scarce. Ordovas et al. [38] showed that the variability in response to dietary manipulation has a genetic component. The genetic variation at specific loci is expected to explain inter-individual variations in lipoprotein response to dietary change. This lipoprotein response to dietary factors seems to be extremely complex, and literature often shows conflicting results [39]. Further research is needed to clarify the variability in (gender-specific) responses of blood lipids to changes in diet quality. Also the potential confounding effect of lifestyle and other factors on blood lipids should be more extensively investigated.

The third main finding was that there were a few differences in results according to the dietary index used, with the MDS showing less longitudinal associations with anthropometric parameters compared to the other two dietary indices. These differences can be due to the type of calculation, with the MDS showing weaker sensitivity because the individual score depends on the median component intakes of the sample. Both cross-sectionally but even more longitudinally the differences in approach and calculation may have an influence on the associations with fitness components. Since the gender-specific median of the MDS was calculated separately for the data gathered in 2002–2004 and 2012–2014, the value of the median might be different between the two time points. This has important implications when change scores are calculated.

The results of the present study are only partly in agreement with cross-sectional studies. Cross-sectional research showed that a low HEI was associated with overweight and obesity in a sample of both men and women [9], while in the present study only an association with anthropometric parameters was found in men. Jovanović et al. [10] found in a cross-sectional study that women having a low HEI score have a two times higher risk to be overweight, while in the present study changes in HEI were not associated with changes in BMI in women. Except for the longitudinal association between the HEI and LDL cholesterol in the unadjusted model in men, no longitudinal associations were found between changes in HEI and changes in blood lipids. Cross-sectional research about the association between the HEI and cardiovascular health parameters mostly showed poor associations [11–13], which is in accordance with the present study. The finding that in the present study there was no longitudinal association between MDS and blood lipids corroborates the results from the Lyon Diet Heart study, in which was found that a Mediterranean diet does not influence the associations between blood lipids and the recurrence rate of CD [16]. In contrast, other cross-sectional studies showed favorable associations with blood lipids [14, 15, 17]. Hoebeeck et al. [19] demonstrated that a higher DQI was cross-sectionally associated with lower triglyceride levels only in the unadjusted model, while in the present study no longitudinal associations with triglycerides were found in both models.

There are some limitations to the present study. The major limitation is the fact that on average the most healthy and interested people returned for retesting. Our drop-out analysis indicated a healthy volunteer effect. Another limitation is that food intake was self-reported by a diet record, which is susceptible to reporting bias [40]. The main strength is the innovative research topic. To our knowledge this is the first study investigating the association between changes in three dietary indices and changes in respectively anthropometric parameters and blood lipids. The longitudinal study design, the investigation of an adult sample with a wide age range, the use of objective measures for anthropometric parameters and blood lipids and the inclusion of potential confounding factors such as an objective parameter for cardiorespiratory fitness [23] are also important strengths.

## Conclusions

It is concluded that only in men an increase in all three dietary indices was associated with an improvement in anthropometric parameters in the non-adjusted analysis and for HEI and DQI also in the adjusted analysis. No longitudinal associations between dietary indices and blood lipids were found in both genders. As this is the first study investigating the association between changes in dietary indices and changes in respectively anthropometric parameters and blood lipids, further research is necessary to investigate this possible relationship.
